# Supplementary material for: Outcome monitoring and risk stratification after cardiac procedure in neonates, infants, children and young adults born with congenital heart disease: protocol for a multicentre prospective cohort study (Children OMACp)
Source: BMJ Open. 2023 Aug 8;13(8):e071629. doi: 10.1136/bmjopen-2023-071629 (PMC10414053; doi:10.1136/bmjopen-2023-071629)
Supplement: Author's manuscript [file bmjopen-2023-071629.draft_revisions.pdf]

BMJ Open is committed to open peer review. As part of this commitment we make the peer review history of every article we publish publicly available.

When an article is published we post the peer reviewers' comments and the authors' responses online. We also post the versions of the paper that were used during peer review. These are the versions that the peer review comments apply to.

The versions of the paper that follow are the versions that were submitted during the peer review process. They are not the versions of record or the final published versions. They should not be cited or distributed as the published version of this manuscript.

BMJ Open is an open access journal and the full, final, typeset and author-corrected version of record of the manuscript is available on our site with no access controls, subscription charges or pay-per-view fees (http://bmjopen.bmj.com).

If you have any questions on BMJ Open's open peer review process please email info.bmjopen@bmj.com

Children OMACp Protocol: A multi-centre prospective cohort study monitoring outcome and risk stratification after cardiac procedure in neonates, infants, children and young adults born with congenital heart disease.

Journal:	*BMJ Open*
Manuscript ID	bmjopen-2023-071629
Article Type:	Protocol
Date Submitted by the Author:	05-Jan-2023
Complete List of Authors:	Baquedano, Mai; University of Bristol; University Hospitals Bristol and Weston NHS Foundation Trust<br>de Jesus, Samantha; University of Bristol<br>Rapetto, Filippo; University Hospitals Bristol and Weston NHS Foundation Trust<br>Murphy, Gavin; NIHR Leicester Biomedical Research Centre Cardiovascular Diseases, Department of Cardiovascular Sciences and NIHR Leicester Biomedical Research Unit in Cardiovascular Medicine<br>Angelini, Gianni; University of Bristol, Bristol Medical School ; University Hospitals Bristol and Weston NHS Foundation Trust<br>Benedetto, Umberto; University Hospitals Bristol and Weston NHS Foundation Trust<br>Caldas, Patricia; University Hospitals Bristol and Weston NHS Foundation Trust<br>Srivastava, Prashant K.; Imperial College London<br>Uzun, Orhan; Cardiff and Vale NHS Trust<br>Luyt, Karen; University of Bristol, Bristol Medical School; University Hospitals Bristol and Weston NHS Foundation Trust, NICU<br>Gonzalez Corcia, Cecilia; University Hospitals Bristol and Weston NHS Foundation Trust<br>Taliotis, Demetris; University Hospitals Bristol and Weston NHS Foundation Trust<br>Stoica, Serban; Bristol Royal Hospital for Children<br>Lawlor, Deborah; University of Bristol, MRC Integrative Epidemiology Unit<br>Bamber, Andrew; North Bristol NHS Trust<br>Perry, Alison; University Hospitals Bristol and Weston NHS Foundation Trust<br>Skeffington, Katie L.; University of Bristol<br>Omeje, Ikenna; University Hospitals of Leicester NHS Trust<br>Pappachan, John; Southampton Children's Hospital, PICU<br>Mumford, Andrew; University Hospitals Bristol and Weston NHS Foundation Trust, Department of Haematology<br>Coward, Richard J.M.; University of Bristol<br>Caputo, Massimo; University of Bristol, Bristol Heart Institute
Keywords:	Congenital heart disease < CARDIOLOGY, Coronary intervention < CARDIOLOGY, Paediatric cardiology < CARDIOLOGY, Valvular heart

	disease < CARDIOLOGY

SCHOLARONE™
Manuscripts

Children OMACp Protocol: A multi-centre prospective cohort study monitoring outcome and risk stratification after cardiac procedure in neonates, infants, children and young adults born with congenital heart disease.

Authors:

M. Baquedano[1,2*], S.E. de Jesus[1], F. Rapetto[2], G.J. Murphy[4], G. Angelini[1,2], U. Benedetto[2], P. Caldas[2], P.K. Srivastava[5], O. Uzun[6], K. Luyt[1,2], C. Gonzalez Corcia[2], D. Taliotis[2], S. Stoica[2], D.A. Lawlor[1,9], A. Bamber[3], A. Perry[2], K.L. Skeffington[12], I. Omeje[7], J. Pappachan[8], A.D. Mumford[10], R.J.M. Coward[1], D. Kenny[11], M. Caputo[1,2]

[1]Bristol Medical School, University of Bristol, Bristol, UK; [2]University Hospitals Bristol and Weston NHS Foundation Trust, Bristol, UK; [3]North Bristol NHS Foundation Trust, Bristol, UK; [4]Department of Cardiovascular Sciences, University of Leicester, Leicester, UK; [5]National Heart & Lung Institute, Imperial College London, London, UK; [6]University Hospital of Wales, Cardiff and Vale NHS Trust, Cardiff, UK; [7]East Midlands Congenital Heart Centre, University Hospitals of Leicester, Leicester, UK; [8]University Hospital Southampton NHS Foundation Trust, Southampton, UK; [9]Medical Research Council Integrative Epidemiology Unit at the University of Bristol, Bristol, UK; [10]School of Cellular and Molecular Medicine, University of Bristol; [11]Children's Health Ireland at Crumlin, Dublin, Ireland; [12]Bristol Heart Institute, University of Bristol , Bristol, UK

Corresponding Author:

*Mai Baquedano, University of Bristol, Bristol Royal Hospital for Children, Upper Maudlin Street, Bristol, BS2 8BJ. Email: mai.baquedano@bristol.ac.uk

Word Count: 3,466

For peer review only - http://bmjopen.bmj.com/site/about/guidelines.xhtml
UOB Confidential

ABSTRACT

Introduction

Congenital heart disease (CHD) represents the most common birth defect, affecting from 0.4 – 1.2% of children born in developed countries. The survival of these patients has increased significantly, but CHD remains one of the major causes of neonatal and childhood death. The aetiology of CHD is complex, with some evidence of both genetic and environmental causes. However, there is still lack of knowledge regarding modifiable risk factors and molecular and genetic mechanisms underlying the development of CHD. This study aims to develop a prospective cohort of patients undergoing cardiac procedures that will bring together routinely collected clinical data and biological samples from patients and their biological mothers, in order to investigate risk factors and predictors of post-operative- outcomes, as well as better understanding the efficacy of the surgical intervention on the early and long-term outcomes.

Methods and Analysis

Children OMACp is a multi-centre, prospective cohort study recruiting children with CHD undergoing a cardiac procedure. The study aims to recruit 3000 participants over 5 years (2019-2024) across multiple UK sites. Routine clinical data will be collected, as well as participant questionnaires collecting sociodemographic, NHS resource use and quality of life data. Biological samples (blood, urine and surgical waste tissue from patients, and blood and urine samples from biological mothers) will be collected where consent has been obtained. Follow up outcome and questionnaire data will be collected for 5 years.

Ethics and Dissemination

The study was approved by the London–Brent Research Ethics Committee on 30/07/2019 (19/SW/0113). The study is sponsored by the University Hospitals of Bristol and Weston Foundation Trust and is managed by the University of Bristol. Children OMACp is adopted onto the National Institute for Health Research Clinical Research Network portfolio. Findings will be disseminated through peer-reviewed publications, presentation at conference, meetings and through patient organisations and newsletters.

Trial registration number:

ISRCTN – 17650644

For peer review only

UOB Confidential

STRENGTHS AND LIMITATIONS

- Large multi-centre, prospective cohort study collecting routine clinical data with biological samples and questionnaire data in children with congenital heart disease undergoing a cardiac procedure based in the UK and Ireland,
- The study also collects data and samples from patient's biological mothers.
- Data and samples analyses will allow identification of risk factors, predictors of short-, medium- and long-term clinical outcomes, as well as providing answers to numerous research questions.
- This study is an efficient and cost-effective method of generating a persistent data and sample resource that can be made accessible both nationally and internationally to underpin future CHD studies.
- The cohort does not include control data from healthy subjects. We will explore obtaining control data from external sources (e.g. Avon Longitudinal Study of Parents and Children (ALSPAC) and record linkage)

For peer review only

UOB Confidential

INTRODUCTION

Congenital heart disease (CHD) represents the most common birth defect, affecting from 0.4 – 1.2% of live-born children in the developed countries [1, 2], and remains one of the major causes of neonatal, infant and childhood death [3, 2]. CHD is generally defined as a gross structural abnormality of the heart or intrathoracic great vessels present at birth that is actually or potentially of functional significance [4].

The aetiology of CHD is complex, with some evidence of both genetic and environmental causes. However, there is still a lack of knowledge regarding modifiable causal risk factors [5]. Furthermore, the molecular and genetic mechanisms underlying the development of CHD have not been clearly elucidated and the identification of specific sub-phenotypes of CHD remains challenging [6, 7].

Whilst the incidence of CHD has remained unchanged, the survival of these neonates and infants has increased dramatically in the last two decades [8], leading to a rise in the numbers of adults with CHD. For example, in 2000, there were nearly equal numbers of adults and children with severe CHD [9], highlighting the need to better understand CHD aetiology, including modifiable risk factors and molecular genetic mechanisms, so that methods of preventing CHD can be developed to reduce its disease burden.[10]

Current research on potentially modifiable maternal risk factors suggests that maternal smoking, greater adiposity and associated dyslipidaemia, gestational diabetes and hypertensive disorders of pregnancy are associated with increased incidence of CHD [11-13]. However, most of these studies have been cross-sectional or case control, with relatively small number of cases or have not adjusted for key confounders and therefore cannot determine degree of causality. Genome-wide association studies (GWAS) of the fetal/child genome including up to 2000 cases have identified some robust genetic variants associated with all or specific CHDs, but lack power to identify modest genetic associations, particularly with sub-types of CHD [14]. GWAS of maternal genotype are rare and few aetiological studies have used methods to try and unpick causal effects from association, such as using genetic variants as instrumental variables in Mendelian randomization studies, negative control studies or within sibship analyses [15]. It is necessary to apply best methods to establish the most relevant targets for interventions in order to facilitate future randomised controlled trials assessing causality.

For peer review only

UOB Confidential

Patients with CHD often require multiple complex surgeries and life-long medical therapies and follow-up. These therapies are ineffective in up to 50% of the treated patients and involve significant morbidity, long-term mortality and substantial cost [16].

The development of predictive tools and integrated information for advancing CHD clinical diagnosis and prognosis is one of the most prominent challenges of clinical cardiology. Traditional research methods have largely failed to advance an understanding of early complications and longer-term failure of cardiac surgery interventions. However, application of large-scale genomic [17] and metabolomic [18, 19] approaches to the prediction of complications and applied epidemiological techniques to investigate causality [20, 21] have the potential to advance mechanistic understanding and streamline therapeutic development [22].

In parallel, Electronic Health Records (EHR) data generated and collected during normal clinical care are increasingly being linked and used for translational cardiovascular research.

Our ability to advance medical care and efficiently translate science into modern medicine is bounded by our capacity to access and process these 'Big Data'. From human genetics and 'omics' to routine clinical documentation and from internal imaging to motion capture, the volume and variety of "Big Data" is bound to change in medicine. Statistical, mathematical, visualization, and computational approaches, from a wide range of disciplines, as well systems for innovative interventions, are needed to keep apace of the complexity in disease prediction and prognostics and to advance medicine.

The study aims to develop a prospective cohort of patients undergoing surgery and/or catheterisation that will bring together routinely collected clinical data as well as biological samples from both patients and their biological mothers, in order to investigate further risk factors of CHD and predictors of post-operative outcomes. Our ambition is to make this a persistent resource accessible to high quality future CHD studies, nationally and internationally.

METHODS AND ANALYSIS

Study design and setting

Children OMACp is a multi-centre, prospective cohort study recruiting patients diagnosed with CHD and undergoing a cardiac procedure (catheterisation and/or surgery) and their biological mothers. The study aims to evaluate short, medium and long-term health outcomes and collect biological samples for patients undergoing cardiac surgery and/or catheterisation for CHD.

For peer review only

UOB Confidential

Biological samples and risk factors information will also be collected from patients' biological mothers where consent has been obtained.

Study Setting

The cohort will recruit patients attending Bristol Royal Hospital for Children, and up to 11 additional hospitals. At the time of writing, the study is recruiting at Bristol Royal Hospital for Children, Leicester Royal Infirmary and Dublin Children's Hospital.

The planned duration of the study is 10 years (5 years recruitment period and 5 years for follow up).

Research Questions

Specific research questions and objectives include, but are not limited to:

a) To identify the maternal genetic and potentially modifiable risk factors for CHD.
b) To identify clinical, genetic and environmental factors that act as predictors for short, medium and long-term clinical outcomes.
c) To determine the optimal clinical management of CHD.
d) To identify factors that influence NHS resource use for patients with CHD.
e) To evaluate the impact of CHD on neurological development and school performance
f) To evaluate the impact of CHD surgical correction on organ function (such as heart, liver, kidney, that are potentially damaged during the operation).
g) To identify the impact of CHD on sleep disorders.
h) To evaluate the accuracy and prediction of imaging cardiac data with early and long-term complications.
i) To use machine learning platforms for developing personalised treatment for CHD (e.g. pharmacogenetics, and epigenetics)

Study population

The target population includes neonates, infants and children (0 to 15 years) and young adults (16 to 18 years) with CHD undergoing catheterisation and/or surgery at participating centres, and their biological mothers. The study aims to recruit 3000 participants over 5 years (2019 – 2024).

For peer review only

UOB Confidential

Inclusion criteria

Patients must:

a) be aged between 0 months and 18 years of age

b) diagnosed with CHD

c) undergoing cardiac surgery and/or catheterisation

The person giving consent must:

d) have capacity to consent (if participant 16 to 18 years of age) or assent (if participant age 11 to 15)

e) have capacity to consent and parental responsibility for the participant (if participant under 16 years of age)

Mothers must:

a) have a biological child enrolled in Children OMACp

b) have capacity to consent

Exclusion criteria

The study will exclude patients and biological mothers who are unable to give informed consent and/or assent, those with a main residence outside of the UK and Ireland and those under the care of Social Services.

Research procedures

Identification of participants and consent

Recruitment will be from September 2019 to August 2024. The majority of patients will be identified by the clinical team from the cardiac surgical/catheter waiting lists. Some patients may be identified on admission, 12-24 hours prior to surgery. All eligible patients, and their biological mother, will be invited to participate in the study. The study is conducted in accordance with the Declaration of Helsinki. All biological samples and questionnaires are optional.

For peer review only

UOB Confidential

Recruitment

Before admission for cardiac surgery and/or catheterisation, participants and/or their parent/guardian, as well as their biological mother when possible, will be provided with participant information leaflets (PILs). These contain a study description including study details, study contacts and details of withdrawal processes. Following a period of at least 12 hours to consider the study, participants who are interested in joining will be asked to provide informed consent. Participants (if 16 or over) or their parent/guardian (if participant age is 0 to 15) will provide consent to routine data collection. Participants and/or parents/guardian can also optionally consent to biological samples being taken, stored and analysed, as well as to complete study questionnaires at baseline and during follow up. Patients who are aged 11-15 years at the time of consent will also be asked to complete an assent form. This assent form will be respected. Participants who turn 16 years old during the study's follow up period will also be asked to sign a consent form to confirm they are happy to continue taking part in the study. Biological mothers will provide consent for their own data and samples, if they so wish. Consent will be collected electronically where possible.

Samples and Questionnaires

Several bio-samples and questionnaire data can be optionally obtained from patients, and their biological mothers, as part of the study protocol.

Patients Samples

Table 1 summarises the samples that will be collected.

Table 1 – Participant Samples

	Time point		
**Surgical patients**	Pre-op	On arrival to PICU	24h post-arrival to PICU
Blood	**Yes**	**Yes**	**Yes**
Urine	**Yes**	**Yes**	**Yes**
PaxGene RNA	**Yes**	**Yes**	**Yes**
Waste tissue	**Yes**	**N/A**	**N/A**
**Catheter patients**			
Blood	**Yes**	**N/A**	**N/A**
Urine	**Yes**	**N/A**	**N/A**
PaxGene RNA	**Yes**	**N/A**	**N/A**
Waste tissue	**N/A**	**N/A**	**N/A**

For peer review only

UOB Confidential

For patients undergoing catheterisation, blood and urine samples will be taken at baseline only, before their procedure. For patients undergoing cardiac surgery, blood and urine samples will be taken pre-operatively, and post-operatively, on arrival to PICU and at 24h after surgery. All blood samples will be taken through lines that are already in place as part of routine care and blood sample volumes will vary depending on participant age/weight from 1ml to 4ml. Total research blood volumes drawn will take into account routinely collected bloods to ensure patient safety is maintained. Participating centres may also agree to collect optional blood samples for RNA/DNA analyses (using PaxGene RNA tubes).

Waste cardiac tissue and fluids that would otherwise be discarded will also be collected from surgical patients, where consent has been obtained.

Blood and urine samples will be transferred to the local laboratory for processing. Samples will then be stored at -80C. Waste tissue samples will be snap frozen in liquid nitrogen and stored at -80C, unless analyses of fresh tissue samples is required for a specific sub-study, in which case, samples will be processed before freezing.

Patients Questionnaires and Follow Up Data

If the patients are 8 years old or older, a quality-of-life questionnaire that combines the KIDSCREEN, CHU9D and EQ5DY will also be completed by the patient or their parent/guardian prior to the procedure (or within a few days post-procedure), at 3-months and 12-month post-procedure, and every year thereafter for up to 5 years.

All patients, or parents on their behalf if under 16 years, will also be asked to complete a general health and resource use questionnaire 12 months post-procedure and every year thereafter for up to 5 years.

In addition to the yearly questionnaires, the patient's medical notes will be reviewed yearly for 5 years post-procedure.

Mothers Samples and Data

Consented mothers will donate blood and urine samples and will complete a baseline questionnaire to provide demographic and risk factor data. Prenatal medical notes will be accessed where possible.

Blood and urine samples will be transferred to the local laboratory for processing. Samples will then be stored at -80C. Table 2 provides a summary of maternal samples to be collected.

For peer review only

UOB Confidential

Table 2 – Mothers Samples

Mothers	Time point<br>Baseline
Blood	**Yes**
Urine	**Yes**
PaxGene RNA	**Yes**

Data collection

Data will be collected from patients' medical notes, hospital databases and hospital episodes statistics (HES) and ethically approved questionnaires. Data will include, but is not limited to demographic, lifestyle, and socioeconomic data, as well as maternal risk factors, intraoperative details, post-operative complications, NHS resource use and quality of life. Results from the analysis of biological samples (e.g. genomics, metabolomics, proteomics, transcriptomics) will also form part of the final dataset.

All data will be handled in line with the General Data Protection Regulations and Good clinical Practice Guidelines. Data will be collected electronically on specifically designed case report forms and entered onto a REDCap database. Data will be stored on secured servers and access to the database will be restricted to authorised staff only.

Analysis

The study will provide a resource for several research questions to be addressed (see above). There are two broad types of analyses that the cohort will be used for: (a) analyses that addresses questions requiring data from patients with CHD only and (b) analyses addressing questions that require a healthy control/comparison group.

A key focus initially will be on prediction and risk stratification, in particular developing accurate tools for predicting those children who are at most risk of adverse outcomes after surgery, and who are therefore likely to benefit from tailored care-packages and more intense monitoring post-surgery.

Sociodemographic, clinical and 'omics' (e.g. genomics, proteomics, epigenomics and metabolomics) data will be considered for inclusion in the prediction models. Machine learning approaches, that allow for a large number of potential predictors to be studied simultaneously, whilst accounting for their correlation structure and potential interactions to be taken into account, will be used to identify a 'top-set' of potential prediction models. We will then

UOB Confidential

consider those models further with respect to discrimination, calibration and clinical utility, selecting those that maximise cost-effectiveness. We will use internal validation procedures but also (with extension of the network beyond Bristol) have the ability to externally validate the prediction tool in cohorts from outside of Bristol.

With addition of maternal data to the study, we will also explore causal maternal risk factors using multivariable regression analyses [23]. We will also contribute data and academic input to genome wide association study consortia to identify both fetal and maternal genetic variants associated with CHD and use those in two-sample Mendelian randomization.

When reporting outcomes, participants will be grouped according to the type of operation and type of CHD. Analyses will be appropriately powered assessing subgroups of the overall cohort for individual research questions.

When relevant, we will explore options for comparison/control groups, including data from studies such as ALSPAC, as well as data linkage. They will provide suitable healthy controls to explore genetic and environmental determinants of CHD.

Patient and Public Involvement (PPI)

A PPI group reviewed the study documents before the initial ethics application was submitted and comments were incorporated as appropriate.

Our Unit has also been leading many PPI initiative within the OMACp project. Our work in this context focuses on: a) conceiving public presentations of collaborative work created with partners including artists, for broad public engagement and raising awareness around CHD; b) devise and hold workshops for engagement with patient audience exploring their narratives of illness; c) developing creative collaborations across and beyond the University, including academic and community partners; d) participating in outreach events; e) studying the dynamics of arts-and-health collaboration.

For peer review only

UOB Confidential

Withdrawal

All participants will be informed of their right to withdraw from the study at any time, without needing to give a reason. Any data and samples already collected about the participant will remain for analysis unless otherwise requested by the participant.

ETHICS AND DISSEMINATION

Ethics

The study was approved by the London – Brent Research Ethics Committee on 30/07/2019 (19/SW/0113) and is adopted onto the National Institute for Health Research Clinical Research Network portfolio. The study is sponsored by the University Hospitals of Bristol and Weston Foundation Trust and is managed by the University of Bristol and has organised indemnification. Protocol deviations will be documented and reported to the chief investigator and the sponsor immediately.

Given this is an observational study that does not change the patient's standard care, there are no risks to patient safety resulting from the study. Child blood samples are taken through pre-existing lines inserted for clinical indications within safe volumes for child weigh. Collecting urine and waste tissue samples does not pose any additional risks. Therefore, it is not possible for clinical adverse events to be attributed to study specific procedures. For mothers, there is a very minimal risk associated with blood withdrawal.

Dissemination

Baseline characteristics of the cohort and any finding will be presented at local, national and international meetings and published in peer-reviewed papers. Findings will also be presented to patients through patient organisations and newsletters.

The resource generated from this study, which includes routine data, samples and questionnaire data, will be made available to other researchers for future studies nationally and internationally. Our aim is to establish this as a persistent resource that will be made accessible to high-quality, ethically approved research by submitting a proposal and access request to be considered by the Children OMACp Executive Group, similar to procedures used by general cohorts such as UK Biobank, ALSPAC and CRANE. A full description of the data and samples available will be accessible through the study's website.

For peer review only

UOB Confidential

SUMMARY

Here we present the Children OMACp study, a unique resource facilitating research into CHD by enabling efficient collection of large amounts of data from medical records and questionnaires, as well as biological samples from multiple hospitals performing heart procedures on this specific cohort of patients.

OMACp has many strengths, providing the ability to develop a cohort of patients with CHD in UK and Ireland, for robust hypothesis driven research and will focus initially on prediction and risk stratification for short-, medium- and long-term outcomes, offering the basis to explore risk, prognosis, outcome and management. The final goal is to create a data registry and biosample repository that can be made available to other high quality ethically approved studies to support future CHD research nationally and internationally.

Combining peri-operative data, maternal risk factors information and biosamples, the study will explore the potential for machine learning and personalised medicine, by utilising artificial intelligence models to review sociodemographic, clinical (including imaging data) and multiple 'omics (e.g. genomic, epigenomic, proteomics and metabolomics) to better risk stratified the extreme large heterogenous diagnosis of CHD and their surgical early- and long-term outcomes and prepare individually tailored approaches to care. We will use data from external sources (e.g. ALSPAC and record linkage) as healthy controls, when necessary. The collection of maternal data will contribute information on the potential causality of maternal risk factors.

This study will further explore the potential of deep phenotyping the extreme high-risk group of patients with single ventricle and cyanotic heart disease, as well as evaluating the long-term impact of CHD treatment on neurocognitive development.

CHANGES TO PROTOCOL SINCE REC APPROVAL

The study began recruiting in September 2019 using version 2 of the protocol. When the study began, it was single-centre, recruiting only in Bristol, and patients only completed quality of life questionnaires at 3- and 12-months post-procedure. In January 2020 the study became multi-centre and in January 2021 yearly general health and quality of life questionnaires were introduced for the 5-year follow up period. The current version of the protocol is version 5 dated 01/03/2022.

For peer review only - http://bmjopen.bmj.com/site/about/guidelines.xhtml

UOB Confidential

FUNDING STATEMENT

The study is funded by the NIHR Bristol Biomedical Research Centre (BRC-1215-20011) and Professor Massimo Caputo's British Heart Foundation Personal Chairs (BHF - (CH/17/1/32804). Deborah A Lawlor's contribution to the study is supported by her British Heart Foundation Chair (CH/F/20/90003) and the MRC Integrative Epidemiology Unit (MC_UU_00011/6). Gavin J Murphy's contribution to the study is supported by the BHF (CH/12/1/29419 and RG/17/9/32812). Ikenna Omeje's contribution to the study is supported by the Heart Link Children's charity.

COMPETING INTERESTS

D.A.L has received support from Medtronic Ltd and Roche Diagnostics for research unrelated to this publication. Other authors have no conflicts of interest to declare.

For peer review only

UOB Confidential

REFERENCES

1. Hoffman JIE, Kaplan S. The incidence of congenital heart disease. *J Am Coll Cardiol*. 2002;39:1890–1900.

2. Hoffman JIE. Incidence, mortality and natural history. In: Anderson R, Baker E, Macartney F, Rigby M, Shinebourne E, Tynan M, editors. *Paediatr Cardiol*. Second. London: Churchill Livingstone; 2002. p. 111–139.

3. Petrini J, Damus K, Russell R, et al. Contribution of birth defects to infant mortality in the United States. Teratology 66, 2002 (Suppl 1): S3–S6.

4. Mitchell SC, Korones SB, Berendes HW. Congenital heart disease in 56,109 births. Incidence and natural history. *Circulation*. 1971;43:323–332.

5. Zhu H, Kartiko S, Finnell RH. Importance of gene–environment interactions in the etiology of selected birth defects. Clin Genet 2009;75:409–23.

6. Gelb BD, Chung WK. Complex genetics and the etiology of human congenital heart disease. Cold Spring Harb Perspect Med 2014;4:a013953.

7. Lalani SR, Belmont JW. Genetic basis of congenital cardiovas- cular malformations. Eur J Med Genet 2014;57:402–13

8. Birth Prevalence of Congenital Heart Disease Worldwide A Systematic Review and Meta-Analysis Denise van der Linde, MSC, Elisabeth E. M. Konings, BSC, Maarten A. Slager, BSC, Maarten Witsenburg, MD, PHD, Willem A. Helbing, MD, PHD, Johanna J. M. Takkenberg, MD, PHD, Jolien W. Roos-Hesselink, MD, PHD

9. Marelli AJ, Mackie AS, Ionescu-Ittu R, Rahme E, Pilote L. Congenital heart disease in the general population. Circulation. 2007; 115:163-172

10. Botto LD, Correa A, Erickson JD. Racial and temporal variations in the prevalence of heart defects. Pediatrics. 2001; 107:e32.

11. Abu-Sulaiman RM, Subaih B. Congenital heart disease in infants of diabetic mothers: echocardiographic study. Pediatr Cardiol. 2004; 25:137-140

12. Alverson CJ, Strickland MJ, Gilboa SM, Correa A. Maternal smoking and congenital heart defects in the Baltimore-Washington infant study. Pediatrics. 2011; 127:e647-e653

13. Cedergren MI, Källén BAJ. Maternal obesity and infant heart defects. Obes Res. 2003; 11:1065-1071

For peer review only

UOB Confidential

14. Agopian AJ, Goldmuntz E, Hakonarson H, Sewda A, Taylor D, Mitchell LE; Pediatric Cardiac Genomics Consortium. Genome-Wide Association Studies and Meta-Analyses for Congenital Heart Defects. Circ Cardiovasc Genet. 2017 Jun;10(3):e001449. doi: 10.1161/CIRCGENETICS.116.001449.

15. Cordell HJ, Bentham J, Topf A, Zelenika D, Heath S, Mamasoula C, Cosgrove C, Blue G, Granados-Riveron J, Setchfield K, Thornborough C, Breckpot J, Soemedi R, Martin R, Rahman TJ, Hall D, van Engelen K, Moorman AF, Zwinderman AH, Barnett P, Koopmann TT, Adriaens ME, Varro A, George AL Jr, dos Remedios C, Bishopric NH, Bezzina CR, O'Sullivan J, Gewillig M, Bu'Lock FA, Winlaw D, Bhattacharya S, Devriendt K, Brook JD, Mulder BJ, Mital S, Postma AV, Lathrop GM, Farrall M, Goodship JA, Keavney BD. Genome-wide association study of multiple congenital heart disease phenotypes identifies a susceptibility locus for atrial septal defect at chromosome 4p16. Nat Genet. 2013 Jul;45(7):822-4. doi: 10.1038/ng.2637. Epub 2013 May 26.

16. Tennant PW, Pearce MS, Bythell M, Rankin J. 20-year survival of children born with congenital anomalies: a population-based study. Lancet 2010;375:649–56.

17. Consortium, U.K., et al., The UK10K project identifies rare variants in health and disease. Nature, 2015. 526(7571): p. 82-90.

18. Drenos, F., et al., Metabolic Characterization of a Rare Genetic Variation Within APOC3 and Its Lipoprotein Lipase-Independent Effects. Circ Cardiovasc Genet, 2016. 9(3): p. 231-9.

19. Timpson, N.J., et al., A rare variant in APOC3 is associated with plasma triglyceride and VLDL levels in Europeans. Nat Commun, 2014. 5: p. 4871.

20. Nordestgaard, B.G., et al., The effect of elevated body mass index on ischemic heart disease risk: causal estimates from a Mendelian randomisation approach. PLoS Med, 2012. 9(5): p. e1001212.

21. Varbo, A., et al., Remnant cholesterol, low-density lipoprotein cholesterol, and blood pressure as mediators from obesity to ischemic heart disease. Circ Res, 2015. 116(4): p. 665-73.

22. Interleukin-6 Receptor Mendelian Randomisation Analysis, C., et al., The interleukin-6 receptor as a target for prevention of coronary heart disease: a mendelian randomisation analysis. Lancet, 2012. 379(9822): p. 1214-24.

AUTHOR CONTRIBUTIONS

MC is the Chief Investigator of the study, has contributed to the design, oversight and conduct of the study, as well as providing funding from his personal BHF chair.

For peer review only

UOB Confidential

MB has contributed to study design and development of the protocol. MB has managed the study set-up and day-to-day management of the study. MB has also designed and managed the data collection systems.

SDJ has contributed to the management of the study as well as writing the first draft of the protocol paper.

All authors have read and approved the final manuscript.

ACKNOWLEDGEMENTS

We are grateful to the patients and families who supported our patient involvement work and to the patients and families that have consented to and already provided data to the study.

For peer review only

UOB Confidential

BMJ Open

Outcome monitoring and risk stratification after cardiac procedure in neonates, infants, children and young adults born with congenital heart disease: protocol for a multi-centre prospective cohort study (Children OMACp)

Journal:	*BMJ Open*
Manuscript ID	bmjopen-2023-071629.R1
Article Type:	Protocol
Date Submitted by the Author:	04-Jul-2023
Complete List of Authors:	Baquedano, Mai; University of Bristol; University Hospitals Bristol and Weston NHS Foundation Trust<br>de Jesus, Samantha; University of Bristol<br>Rapetto, Filippo; University Hospitals Bristol and Weston NHS Foundation Trust<br>Murphy, Gavin; NIHR Leicester Biomedical Research Centre Cardiovascular Diseases, Department of Cardiovascular Sciences and NIHR Leicester Biomedical Research Unit in Cardiovascular Medicine<br>Angelini, Gianni; University of Bristol, Bristol Medical School ; University Hospitals Bristol and Weston NHS Foundation Trust<br>Benedetto, Umberto; University Hospitals Bristol and Weston NHS Foundation Trust<br>Caldas, Patricia; University Hospitals Bristol and Weston NHS Foundation Trust<br>Srivastava, Prashant K.; Imperial College London<br>Uzun, Orhan; Cardiff and Vale NHS Trust<br>Luyt, Karen; University of Bristol, Bristol Medical School; University Hospitals Bristol and Weston NHS Foundation Trust, NICU<br>Gonzalez Corcia, Cecilia; University Hospitals Bristol and Weston NHS Foundation Trust<br>Taliotis, Demetris; University Hospitals Bristol and Weston NHS Foundation Trust<br>Stoica, Serban; Bristol Royal Hospital for Children<br>Lawlor, Deborah; University of Bristol, MRC Integrative Epidemiology Unit<br>Bamber, Andrew; North Bristol NHS Trust<br>Perry, Alison; University Hospitals Bristol and Weston NHS Foundation Trust<br>Skeffington, Katie L.; University of Bristol<br>Omeje, Ikenna; University Hospitals of Leicester NHS Trust<br>Pappachan, John; Southampton Children's Hospital, PICU<br>Mumford, Andrew; University Hospitals Bristol and Weston NHS Foundation Trust, Department of Haematology<br>Coward, Richard J.M.; University of Bristol<br>Kenny, Damien; Children’s Health Ireland at Crumlin, Dublin<br>Caputo, Massimo; University of Bristol, Bristol Heart Institute
Primary Subject	Cardiovascular medicine

Heading:	
Secondary Subject Heading:	Paediatrics
Keywords:	Congenital heart disease < CARDIOLOGY, Coronary intervention < CARDIOLOGY, Paediatric cardiology < CARDIOLOGY, Valvular heart disease < CARDIOLOGY

SCHOLARONE™
Manuscripts

Outcome monitoring and risk stratification after cardiac procedure in neonates, infants, children and young adults born with congenital heart disease: protocol for a multi-centre prospective cohort study (Children OMACp)

M. Baquedano[1,2*], S.E. de Jesus[1], F. Rapetto[2], G.J. Murphy[4], G. Angelini[1,2], U. Benedetto[2], P. Caldas[2], P.K. Srivastava[5], O. Uzun[6], K. Luyt[1,2], C. Gonzalez Corcia[2], D. Taliotis[2], S. Stoica[2], D.A. Lawlor[1,9], A. Bamber[3], A. Perry[2], K.L. Skeffington[12], I. Omeje[7], J. Pappachan[8], A.D. Mumford[10], R.J.M. Coward[1], D. Kenny[11], M. Caputo[1,2]

[1]Bristol Medical School, University of Bristol, Bristol, UK; [2]University Hospitals Bristol and Weston NHS Foundation Trust, Bristol, UK; [3]North Bristol NHS Foundation Trust, Bristol, UK; [4]Department of Cardiovascular Sciences, University of Leicester, Leicester, UK; [5]National Heart & Lung Institute, Imperial College London, London, UK; [6]University Hospital of Wales, Cardiff and Vale NHS Trust, Cardiff, UK; [7]East Midlands Congenital Heart Centre, University Hospitals of Leicester, Leicester, UK; [8]University Hospital Southampton NHS Foundation Trust, Southampton, UK; [9]Medical Research Council Integrative Epidemiology Unit at the University of Bristol, Bristol, UK; [10]School of Cellular and Molecular Medicine, University of Bristol; [11]Children's Health Ireland at Crumlin, Dublin, Ireland; [12]Bristol Heart Institute, University of Bristol , Bristol, UK

*Correspondence to:

Mai Baquedano

University of Bristol, Bristol Royal Hospital for Children, Upper Maudlin Street, Bristol, BS2 8BJ, UK

Email: mai.baquedano@bristol.ac.uk

Word Count: 3,945

UOB Confidential

ABSTRACT

Introduction

Congenital heart disease (CHD) represents the most common birth defect, affecting from 0.4 – 1.2% of children born in developed countries. The survival of these patients has increased significantly, but CHD remains one of the major causes of neonatal and childhood death. The aetiology of CHD is complex, with some evidence of both genetic and environmental causes. However, there is still lack of knowledge regarding modifiable risk factors and molecular and genetic mechanisms underlying the development of CHD. This study aims to develop a prospective cohort of patients undergoing cardiac procedures that will bring together routinely collected clinical data and biological samples from patients and their biological mothers, in order to investigate risk factors and predictors of post-operative- outcomes, as well as better understanding the effect of the surgical intervention on the early and long-term outcomes.

Methods and analysis

Children OMACp is a multi-centre, prospective cohort study recruiting children with CHD undergoing a cardiac procedure. The study aims to recruit 3000 participants over 5 years (2019-2024) across multiple UK sites. Routine clinical data will be collected, as well as participant questionnaires collecting sociodemographic, NHS resource use and quality of life data. Biological samples (blood, urine and surgical waste tissue from patients, and blood and urine samples from biological mothers) will be collected where consent has been obtained. Follow up outcome and questionnaire data will be collected for 5 years.

Ethics and dissemination

The study was approved by the London–Brent Research Ethics Committee on 30/07/2019 (19/SW/0113). Participants (or their parent/guardian if under 16 years of age) must provide informed consent prior to being recruited into the study. Mothers that wish to take part must also provide informed consent prior to being recruited. The study is sponsored by the University Hospitals of Bristol and Weston Foundation Trust and is managed by the University of Bristol. Children OMACp is adopted onto the National Institute for Health Research Clinical Research Network portfolio. Findings will be disseminated through peer-reviewed publications, presentation at conference, meetings and through patient organisations and newsletters.

Study registration number

For peer review only

UOB Confidential

ISRCTN17650644.

STRENGTHS AND LIMITATIONS OF THIS STUDY

- Large multi-centre, prospective cohort study collecting clinical/questionnaire data and biosamples from children with congenital heart disease (CHD) undergoing cardiac procedure and their biological mothers.
- Combined data and samples analyses will allow identification of risk factors and short-, medium- and long-term outcome predictors.
- Cost-effective method of generating a persistent data and sample registry accessible both nationally and internationally.
- The cohort does not include control data from healthy subjects; however, this will be obtained from external sources and record linkage.
- Only patients requiring a cardiac procedure are included, not representing all CHDs.

review only

UOB Confidential

INTRODUCTION

Congenital heart disease (CHD) represents the most common birth defect, affecting from 0.4 – 1.2% of live-born children in the developed countries [1, 2], and remains one of the major causes of neonatal, infant and childhood death [3, 2]. CHD is generally defined as a gross structural abnormality of the heart or intrathoracic great vessels present at birth that is actually or potentially of functional significance [4].

The aetiology of CHD is complex, with some evidence of both genetic and environmental causes. However, there is still a lack of knowledge regarding modifiable causal risk factors [5]. Furthermore, the molecular and genetic mechanisms underlying the development of CHD have not been clearly elucidated and the identification of specific sub-phenotypes of CHD remains challenging [6, 7].

Whilst the incidence of CHD has remained unchanged, the survival of these neonates and infants has increased dramatically in the last two decades [8], leading to a rise in the numbers of adults with CHD. For example, in 2000, there were nearly equal numbers of adults and children with severe CHD [9], highlighting the need to better understand CHD aetiology, including modifiable risk factors and molecular genetic mechanisms, so that methods of preventing CHD can be developed to reduce its disease burden.[10]

Current research on potentially modifiable maternal risk factors suggests that maternal smoking, greater adiposity and associated dyslipidaemia, gestational diabetes and hypertensive disorders of pregnancy are associated with increased incidence of CHD [11-13]. However, most of these studies have been cross-sectional or case control, with relatively small number of cases or have not adjusted for key confounders and therefore cannot determine degree of causality. Genome-wide association studies (GWAS) of the fetal/child genome including up to 2000 cases have identified some robust genetic variants associated with all or specific CHDs, but lack power to identify modest genetic associations, particularly with sub-types of CHD [14]. GWAS of maternal genotype are rare and few aetiological studies have used methods to try and unpick causal effects from association, such as using genetic variants as instrumental variables in Mendelian randomization studies, negative control studies or within sibship analyses [15]. It is necessary to apply best methods to establish the most relevant targets for interventions in order to facilitate future randomised controlled trials assessing causality.

For peer review only

UOB Confidential

Patients with CHD often require multiple complex surgeries and life-long medical therapies and follow-up. These therapies are ineffective in up to 50% of the treated patients and involve significant morbidity, long-term mortality and substantial cost [16].

The development of predictive tools and integrated information for advancing CHD clinical diagnosis and prognosis is one of the most prominent challenges of clinical cardiology. Traditional research methods have largely failed to advance an understanding of early complications and longer-term failure of cardiac surgery interventions. However, application of large-scale genomic [17] and metabolomic [18, 19] approaches to the prediction of complications and applied epidemiological techniques to investigate causality [20, 21] have the potential to advance mechanistic understanding and streamline therapeutic development [22].

In parallel, Electronic Health Records (EHR) data generated and collected during normal clinical care are increasingly being linked and used for translational cardiovascular research.

Our ability to advance medical care and efficiently translate science into modern medicine is bounded by our capacity to access and process these 'Big Data'. From human genetics and 'omics' to routine clinical documentation and from internal imaging to motion capture, the volume and variety of "Big Data" is bound to change in medicine. Statistical, mathematical, visualization, and computational approaches, from a wide range of disciplines, as well systems for innovative interventions, are needed to keep apace of the complexity in disease prediction and prognostics and to advance medicine.

The study aims to develop a prospective cohort of patients undergoing surgery and/or catheterisation that will bring together routinely collected clinical data as well as biological samples from both patients and their biological mothers, in order to investigate further risk factors of CHD and predictors of post-operative outcomes. Our ambition is to make this a persistent resource accessible to high quality future CHD studies, nationally and internationally. Outcomes and the corresponding data and biomarkers to be examined will be directed by the hypothesis of sub-studies and/or external ethically approved studies accessing the registry.

METHODS AND ANALYSIS

Study design

Children OMACp is a multi-centre, prospective cohort study recruiting patients diagnosed with CHD and undergoing a cardiac procedure (catheterisation and/or surgery) and their biological

For peer review only

UOB Confidential

mothers. The study aims to evaluate short, medium and long-term health outcomes and collect biological samples for patients undergoing cardiac surgery and/or catheterisation for CHD. Biological samples and risk factors information will also be collected from patients' biological mothers where consent has been obtained.

Study setting

The cohort will recruit patients attending Bristol Royal Hospital for Children, and up to 11 additional hospitals. At the time of writing, the study is recruiting at Bristol Royal Hospital for Children, Leicester Royal Infirmary and Dublin Children's Hospital.

The planned duration of the study is 10 years (5 years recruitment period and 5 years for follow up).

Research questions

Specific research questions and objectives include, but are not limited to:

a) To identify the maternal genetic and potentially modifiable risk factors for CHD.
b) To identify clinical, genetic and environmental factors that act as predictors for short, medium and long-term clinical outcomes.
c) To determine the effect of clinical management strategies on outcomes of CHD including peri-operative and operative care.
d) To identify factors that influence NHS resource use for patients with CHD.
e) To evaluate the impact of CHD on neurological development and school performance
f) To evaluate the impact of CHD surgical correction on organ function (such as heart, liver, kidney, that are potentially damaged during the operation).
g) To explore the potential of deep phenotyping extreme high-risk groups including those with single ventricle and cyanotic heart disease
h) To identify the impact of CHD on sleep disorders.
i) To evaluate the accuracy and prediction of imaging cardiac data with early and long-term complications.
j) To use machine learning platforms for developing personalised treatment for CHD (e.g. pharmacogenetics, and epigenetics)

Study population

For peer review only

UOB Confidential

The target population includes neonates, infants and children (0 to 15 years) and young adults (16 to 18 years) with CHD undergoing catheterisation and/or surgery at participating centres, and their biological mothers. The study aims to recruit 3000 participants over 5 years (2019 – 2024).

Inclusion criteria

Patients must:

a) be aged between 0 months and 18 years of age

b) diagnosed with CHD

c) undergoing cardiac surgery and/or catheterisation

The person giving consent must:

d) have capacity to consent (if participant 16 to 18 years of age) or assent (if participant age 11 to 15)

e) have capacity to consent and parental responsibility for the participant (if participant under 16 years of age)

Mothers must:

a) have a biological child enrolled in Children OMACp

b) have capacity to consent

Exclusion criteria

The study will exclude patients and biological mothers who are unable to give informed consent and/or assent, those with a main residence outside of the UK and Ireland and those under the care of Social Services.

Research procedures

Identification of participants and consent

Recruitment will be from September 2019 to August 2024. The majority of patients will be identified by the clinical team from the cardiac surgical/catheter waiting lists. Some patients may be identified on admission, 12-24 hours prior to surgery. All eligible patients, and their biological mother, will be invited to participate in the study. The study is conducted in

For peer review only

UOB Confidential

accordance with the Declaration of Helsinki. All biological samples and questionnaires are optional.

Recruitment

Before admission for cardiac surgery and/or catheterisation, participants and/or their parent/guardian, as well as their biological mother when possible, will be provided with participant information leaflets (PILs). These contain a study description including study details, study contacts and details of withdrawal processes. Following a period of at least 12 hours to consider the study, participants who are interested in joining will be asked to provide informed consent. Participants (if 16 or over) or their parent/guardian (if participant age is 0 to 15) will provide consent to routine data collection. Participants and/or parents/guardian can also optionally consent to biological samples being taken, stored and analysed, as well as to complete study questionnaires at baseline and during follow up. Patients who are aged 11-15 years at the time of consent will also be asked to complete an assent form. This assent form will be respected. Participants who turn 16 years old during the study's follow up period will also be asked to sign a consent form to confirm they are happy to continue taking part in the study. Biological mothers will provide consent for their own data and samples, if they so wish. Consent will be collected electronically where possible.

Samples and questionnaires

Several biosamples and questionnaire data can be optionally obtained from patients, and their biological mothers, as part of the study protocol.

Patient samples

Table 1 summarises the samples that will be collected.

Table 1. Participant samples

	Time point		
**Surgical patients**	Pre-op	On arrival to PICU	24h post-arrival to PICU
Blood	**Yes**	**Yes**	**Yes**
Urine	**Yes**	**Yes**	**Yes**
PaxGene RNA	**Yes**	**Yes**	**Yes**
Waste tissue	**Yes**	**N/A**	**N/A**
**Catheter patients**			
Blood	**Yes**	**N/A**	**N/A**
Urine	**Yes**	**N/A**	**N/A**
PaxGene RNA	**Yes**	**N/A**	**N/A**

For peer review only

UOB Confidential

Waste tissue	**N/A**	**N/A**	**N/A**

For patients undergoing catheterisation, blood and urine samples will be taken at baseline only, before their procedure. For patients undergoing cardiac surgery, blood and urine samples will be taken pre-operatively, and post-operatively, on arrival to PICU and at 24h after surgery. All blood samples will be taken through lines that are already in place as part of routine care and blood sample volumes will vary depending on participant age/weight from 1ml to 4ml. Total research blood volumes drawn will take into account routinely collected bloods to ensure patient safety is maintained. Participating centres may also agree to collect optional blood samples for RNA/DNA analyses (using PaxGene RNA tubes).

Waste cardiac tissue and fluids that would otherwise be discarded will also be collected from surgical patients, where consent has been obtained.

Blood and urine samples will be transferred to the local laboratory for processing. Samples will then be stored at -80C. Waste tissue samples will be snap frozen in liquid nitrogen and stored at -80C, unless analyses of fresh tissue samples is required for a specific sub-study, in which case, samples will be processed before freezing.

Patient questionnaires and follow-up data

If the patients are 8 years old or older, a quality-of-life questionnaire that combines the KIDSCREEN, CHU9D and EQ5DY will also be completed by the patient or their parent/guardian prior to the procedure (or within a few days post-procedure), at 3-months and 12-month post-procedure, and every year thereafter for up to 5 years.

All patients, or parents on their behalf if under 16 years, will also be asked to complete a general health and resource use questionnaire 12 months post-procedure and every year thereafter for up to 5 years.

In addition to the yearly questionnaires, the patient's medical notes will be reviewed yearly for 5 years post-procedure.

Mothers' samples and data

Consented mothers will donate blood and urine samples and will complete a baseline questionnaire to provide demographic and risk factor data. Prenatal medical notes will be accessed where possible.

For peer review only

UOB Confidential

Blood and urine samples will be transferred to the local laboratory for processing. Samples will then be stored at -80C. Table 2 provides a summary of maternal samples to be collected.

Table 2. Mothers' samples

Mothers	Time point<br>Baseline
Blood	**Yes**
Urine	**Yes**
PaxGene RNA	**Yes**

Data collection

Data will be collected from patients' medical notes, hospital databases and hospital episodes statistics (HES) and ethically approved questionnaires. Data will include, but is not limited to demographic, lifestyle, and socioeconomic data, as well as maternal risk factors, intraoperative details, post-operative complications, NHS resource use and quality of life. Results from the analysis of biological samples (e.g. genomics, metabolomics, proteomics, transcriptomics) will also form part of the final dataset.

All data will be handled in line with the General Data Protection Regulations and Good clinical Practice Guidelines. Data will be collected electronically on specifically designed case report forms and entered onto a REDCap database. Data will be stored on secured servers and access to the database will be restricted to authorised staff only.

Analysis

The study will provide a resource for several research questions to be addressed (see above). There are two broad types of analyses that the cohort will be used for: (a) analyses that addresses questions requiring data from patients with CHD only and (b) analyses addressing questions that require a healthy control/comparison group.

A key focus initially will be on prediction and risk stratification, in particular developing accurate tools for predicting those children who are at most risk of adverse outcomes after surgery, and who are therefore likely to benefit from tailored care-packages and more intense monitoring post-surgery.

Sociodemographic, clinical and 'omics' (e.g. genomics, proteomics, epigenomics and metabolomics) data will be considered for inclusion in the prediction models. Machine learning approaches, that allow for a large number of potential predictors to be studied simultaneously,

For peer review only - http://bmjopen.bmj.com/site/about/guidelines.xhtml

UOB Confidential

whilst accounting for their correlation structure and potential interactions to be taken into account, will be used to identify a 'top-set' of potential prediction models. We will then consider those models further with respect to discrimination, calibration and clinical utility, selecting those that maximise cost-effectiveness. We will use internal validation procedures but also (with extension of the network beyond Bristol) have the ability to externally validate the prediction tool in cohorts from outside of Bristol.

With addition of maternal data to the study, we will also explore causal maternal risk factors using multivariable regression analyses. We will also contribute data and academic input to genome wide association study consortia to identify both fetal and maternal genetic variants associated with CHD and use those in two-sample Mendelian randomization.

When reporting outcomes, participants will be grouped according to the type of operation and type of CHD. The children OMACp cohort allows for the collection of routine clinical operative and peri-operative data alongside short (e.g. acute kidney injury) and long term (e.g. mortality) clinical outcomes. We will utilise data and biosamples to answer hypothesis driven research questions such as assessing the effect of CPB duration on renal injury. Analyses will be appropriately powered assessing subgroups of the overall cohort for individual research questions.

When relevant, we will explore options for comparison/control groups, including data from studies such as ALSPAC, as well as data linkage. They will provide suitable healthy controls to explore genetic and environmental determinants of CHD.

Patient and public involvement

A Patient and Public Involvement (PPI) group reviewed the study documents before the initial ethics application was submitted and comments were incorporated as appropriate.

Our Unit has also been leading many PPI initiative within the OMACp project. Our work in this context focuses on: a) conceiving public presentations of collaborative work created with partners including artists, for broad public engagement and raising awareness around CHD; b) devise and hold workshops for engagement with patient audience exploring their narratives of illness; c) developing creative collaborations across and beyond the University, including

For peer review only

UOB Confidential

academic and community partners; d) participating in outreach events; e) studying the dynamics of arts-and-health collaboration.

Withdrawal

All participants will be informed of their right to withdraw from the study at any time, without needing to give a reason. Any data and samples already collected about the participant will remain for analysis unless otherwise requested by the participant.

ETHICS AND DISSEMINATION

Ethics

The study was approved by the London – Brent Research Ethics Committee on 30/07/2019 (19/SW/0113) and is adopted onto the National Institute for Health Research Clinical Research Network portfolio. The study is sponsored by the University Hospitals of Bristol and Weston Foundation Trust and is managed by the University of Bristol and has organised indemnification. Protocol deviations will be documented and reported to the chief investigator and the sponsor immediately.

Given this is an observational study that does not change the patient's standard care, there are no risks to patient safety resulting from the study. Child blood samples are taken through pre-existing lines inserted for clinical indications within safe volumes for child weigh. Collecting urine and waste tissue samples does not pose any additional risks. Therefore, it is not possible for clinical adverse events to be attributed to study specific procedures. For mothers, there is a very minimal risk associated with blood withdrawal. Participants (or their parent/guardian if under 16 years of age) must provide informed consent prior to being recruited into the study. Mothers that wish to take part must also provide informed consent prior to being recruited.

Dissemination

Baseline characteristics of the cohort and any finding will be presented at local, national and international meetings and published in peer-reviewed papers. Findings will also be presented to patients through patient organisations and newsletters.

The resource generated from this study, which includes routine data, samples and questionnaire data, will be made available to other researchers for future studies nationally and internationally. Our aim is to establish this as a persistent resource that will be made accessible to high-quality, ethically approved research by submitting a proposal and access

For peer review only

UOB Confidential

request to be considered by the Children OMACp Executive Group, similar to procedures used by general cohorts such as UK Biobank, ALSPAC and CRANE. A full description of the data and samples available will be accessible through the study's website.

Changes to protocol since ethical approval

The study began recruiting in September 2019 using version 2 of the protocol. When the study began, it was single-centre, recruiting only in Bristol, and patients only completed quality of life questionnaires at 3- and 12-months post-procedure. In January 2020 the study became multi-centre and in January 2021 yearly general health and quality of life questionnaires were introduced for the 5-year follow up period. The current version of the protocol is version 5 dated 01/03/2022.

DISCUSSION

Here we present the Children OMACp study, a unique resource facilitating research into CHD by enabling efficient collection of large amounts of data from medical records and questionnaires, as well as biological samples from multiple hospitals performing heart procedures on this specific cohort of patients and their biological mothers.

OMACp has many strengths, providing the ability to develop a cohort of patients with CHD in UK and Ireland, for robust hypothesis driven research and will focus initially on identification of risk factors and outcome predictors for short-, medium- and long-term outcomes, offering the basis to explore risk, prognosis, outcome and management. The final goal is to create a cost-effective, persistent, data and biosample repository that can be made available to other high quality ethically approved studies to support future CHD research nationally and internationally.

Combining peri-operative data, maternal risk factors information and biosamples, the study will explore the potential for machine learning and personalised medicine, by utilising artificial intelligence models to review sociodemographic, clinical (including imaging data) and multiple ‘omics (e.g. genomic, epigenomic, proteomics and metabolomics) to better risk stratified the extreme large heterogenous diagnosis of CHD and their surgical early- and long-term outcomes and prepare individually tailored approaches to care. The collection of maternal data will contribute information on the potential causality of maternal risk factors.

For peer review only

UOB Confidential

Whilst initially, the study will collect only minimal information on father's history (via the mother's questionnaire), in future, the aim is to extend the recruitment to fathers to assess impact of father's genetics, medical and lifestyle factors on children CHD risk and outcomes. Furthermore, there will be potential for including additional non-cardiac malformations.

Although only patients requiring a cardiac procedure are included, representing a fraction of all CHDs, this is the group that will most greatly benefit from any advances in the management and tailoring of medical care.

In addition, whilst the cohort does not include healthy subjects, the study has access to use data and samples from external sources (e.g. ALSPAC and record linkage) as healthy controls, when necessary.

This study will further explore the potential of deep phenotyping extreme high-risk groups, including those with single ventricle and cyanotic heart disease, as well as evaluating the long-term impact of CHD treatment on neurocognitive development

*** ***

FUNDING

The study is funded by the NIHR Bristol Biomedical Research Centre (BRC-1215-20011) and Professor Massimo Caputo's British Heart Foundation Personal Chairs (BHF - (CH/17/1/32804). Deborah A Lawlor's contribution to the study is supported by her British Heart Foundation Chair (CH/F/20/90003) and the MRC Integrative Epidemiology Unit (MC_UU_00011/6). Gavin J Murphy's contribution to the study is supported by the BHF (CH/12/1/29419 and RG/17/9/32812). Ikenna Omeje's contribution to the study is supported by the Heart Link Children's charity.

COMPETING INTERESTS

D.A.L has received support from Medtronic Ltd and Roche Diagnostics for research unrelated to this publication. Other authors have no conflicts of interest to declare.

CONTRIBUTORS

For peer review only

UOB Confidential

MC and MB conceptualised the project. MB, MC, DAL and UB developed the protocol. FR, GJM, GA, UB, PC, PKS, OU, KL,CGC, DT, SS, DAL, AB, AP, KLS, IO, JP, ADM, RJMC, DK approved protocol design. MB, SEdJ contributed to the oversight and conduct of the study. MB designed and managed the data collection systems. MC, GJM, DAL and IO contributed to the funding of the study. SEdJ and MB drafted the study protocol manuscript and received comments from all coauthors. All authors have read and approved the final manuscript.

ACKNOWLEDGEMENTS

We are grateful to the patients and families who supported our patient involvement work and to the patients and families that have consented to and already provided data to the study.

For peer review only

UOB Confidential

REFERENCES

1. Hoffman JIE, Kaplan S. The incidence of congenital heart disease. *J Am Coll Cardiol*. 2002;39:1890–1900.

2. Hoffman JIE. Incidence, mortality and natural history. In: Anderson R, Baker E, Macartney F, Rigby M, Shinebourne E, Tynan M, editors. *Paediatr Cardiol*. Second. London: Churchill Livingstone; 2002. p. 111–139.

3. Petrini J, Damus K, Russell R, et al. Contribution of birth defects to infant mortality in the United States. Teratology 66, 2002 (Suppl 1): S3–S6.

4. Mitchell SC, Korones SB, Berendes HW. Congenital heart disease in 56,109 births. Incidence and natural history. *Circulation*. 1971;43:323–332.

5. Zhu H, Kartiko S, Finnell RH. Importance of gene–environment interactions in the etiology of selected birth defects. Clin Genet 2009;75:409–23.

6. Gelb BD, Chung WK. Complex genetics and the etiology of human congenital heart disease. Cold Spring Harb Perspect Med 2014;4:a013953.

7. Lalani SR, Belmont JW. Genetic basis of congenital cardiovas- cular malformations. Eur J Med Genet 2014;57:402–13

8. Birth Prevalence of Congenital Heart Disease Worldwide A Systematic Review and Meta-Analysis Denise van der Linde, MSC, Elisabeth E. M. Konings, BSC, Maarten A. Slager, BSC, Maarten Witsenburg, MD, PHD, Willem A. Helbing, MD, PHD, Johanna J. M. Takkenberg, MD, PHD, Jolien W. Roos-Hesselink, MD, PHD

9. Marelli AJ, Mackie AS, Ionescu-Ittu R, Rahme E, Pilote L. Congenital heart disease in the general population. Circulation. 2007; 115:163-172

10. Botto LD, Correa A, Erickson JD. Racial and temporal variations in the prevalence of heart defects. Pediatrics. 2001; 107:e32.

11. Abu-Sulaiman RM, Subaih B. Congenital heart disease in infants of diabetic mothers: echocardiographic study. Pediatr Cardiol. 2004; 25:137-140

12. Alverson CJ, Strickland MJ, Gilboa SM, Correa A. Maternal smoking and congenital heart defects in the Baltimore-Washington infant study. Pediatrics. 2011; 127:e647-e653

13. Cedergren MI, Källén BAJ. Maternal obesity and infant heart defects. Obes Res. 2003; 11:1065-1071

For peer review only

UOB Confidential

14. Agopian AJ, Goldmuntz E, Hakonarson H, Sewda A, Taylor D, Mitchell LE; Pediatric Cardiac Genomics Consortium. Genome-Wide Association Studies and Meta-Analyses for Congenital Heart Defects. Circ Cardiovasc Genet. 2017 Jun;10(3):e001449. doi: 10.1161/CIRCGENETICS.116.001449.

15. Cordell HJ, Bentham J, Topf A, Zelenika D, Heath S, Mamasoula C, Cosgrove C, Blue G, Granados-Riveron J, Setchfield K, Thornborough C, Breckpot J, Soemedi R, Martin R, Rahman TJ, Hall D, van Engelen K, Moorman AF, Zwinderman AH, Barnett P, Koopmann TT, Adriaens ME, Varro A, George AL Jr, dos Remedios C, Bishopric NH, Bezzina CR, O'Sullivan J, Gewillig M, Bu'Lock FA, Winlaw D, Bhattacharya S, Devriendt K, Brook JD, Mulder BJ, Mital S, Postma AV, Lathrop GM, Farrall M, Goodship JA, Keavney BD. Genome-wide association study of multiple congenital heart disease phenotypes identifies a susceptibility locus for atrial septal defect at chromosome 4p16. Nat Genet. 2013 Jul;45(7):822-4. doi: 10.1038/ng.2637. Epub 2013 May 26.

16. Tennant PW, Pearce MS, Bythell M, Rankin J. 20-year survival of children born with congenital anomalies: a population-based study. Lancet 2010;375:649–56.

17. Consortium, U.K., et al., The UK10K project identifies rare variants in health and disease. Nature, 2015. 526(7571): p. 82-90.

18. Drenos, F., et al., Metabolic Characterization of a Rare Genetic Variation Within APOC3 and Its Lipoprotein Lipase-Independent Effects. Circ Cardiovasc Genet, 2016. 9(3): p. 231-9.

19. Timpson, N.J., et al., A rare variant in APOC3 is associated with plasma triglyceride and VLDL levels in Europeans. Nat Commun, 2014. 5: p. 4871.

20. Nordestgaard, B.G., et al., The effect of elevated body mass index on ischemic heart disease risk: causal estimates from a Mendelian randomisation approach. PLoS Med, 2012. 9(5): p. e1001212.

21. Varbo, A., et al., Remnant cholesterol, low-density lipoprotein cholesterol, and blood pressure as mediators from obesity to ischemic heart disease. Circ Res, 2015. 116(4): p. 665-73.

22. Interleukin-6 Receptor Mendelian Randomisation Analysis, C., et al., The interleukin-6 receptor as a target for prevention of coronary heart disease: a mendelian randomisation analysis. Lancet, 2012. 379(9822): p. 1214-24.

For peer review only

UOB Confidential
